# Role of Transposon-Derived Small RNAs in the Interplay between Genomes and Parasitic DNA in Rice

**DOI:** 10.1371/journal.pgen.1002953

**Published:** 2012-09-27

**Authors:** Misuzu Nosaka, Jun-Ichi Itoh, Yasuo Nagato, Akemi Ono, Aiko Ishiwata, Yutaka Sato

**Affiliations:** 1 Department of Biological Mechanisms and Functions, Graduate School of Bioagricultural Sciences, Nagoya University, Nagoya, Japan; 2 Department of Agricultural and Environmental Biology, Graduate School of Agricultural and Life Sciences, University of Tokyo, Tokyo, Japan; 3 PRESTO, Japan Science and Technology Agency (JST), Kawaguchi, Japan; University of Cambridge, United Kingdom

## Abstract

RNA silencing is a defense system against “genomic parasites” such as transposable elements (TE), which are potentially harmful to host genomes. In plants, transcripts from TEs induce production of double-stranded RNAs (dsRNAs) and are processed into small RNAs (small interfering RNAs, siRNAs) that suppress TEs by RNA–directed DNA methylation. Thus, the majority of TEs are epigenetically silenced. On the other hand, most of the eukaryotic genome is composed of TEs and their remnants, suggesting that TEs have evolved countermeasures against host-mediated silencing. Under some circumstances, TEs can become active and increase in copy number. Knowledge is accumulating on the mechanisms of TE silencing by the host; however, the mechanisms by which TEs counteract silencing are poorly understood. Here, we show that a class of TEs in rice produces a microRNA (miRNA) to suppress host silencing. Members of the *microRNA820* (*miR820*) gene family are located within CACTA DNA transposons in rice and target a *de novo* DNA methyltransferase gene, *OsDRM2*, one of the components of epigenetic silencing. We confirmed that *miR820* negatively regulates the expression of *OsDRM2*. In addition, we found that expression levels of various TEs are increased quite sensitively in response to decreased *OsDRM2* expression and DNA methylation at TE loci. Furthermore, we found that the nucleotide sequence of *miR820* and its recognition site within the target gene in some *Oryza* species have co-evolved to maintain their base-pairing ability. The co-evolution of these sequences provides evidence for the functionality of this regulation. Our results demonstrate how parasitic elements in the genome escape the host's defense machinery. Furthermore, our analysis of the regulation of *OsDRM2* by *miR820* sheds light on the action of transposon-derived small RNAs, not only as a defense mechanism for host genomes but also as a regulator of interactions between hosts and their parasitic elements.

## Author Summary

Transposons, which are sometimes referred to as “genomic parasites,” are a major component of eukaryotic genomes. Because transposon activity is often detrimental to host genome stability, most transposons are silenced by the host's defense machinery. The mechanisms of transposon silencing, such as RNA silencing, have been well investigated, but virtually nothing is known about the strategies that transposons have evolved to avoid silencing. In this paper, we demonstrate that a microRNA (miRNA) produced from a transposon suppresses the host's silencing machinery. Generally, small interfering RNAs (siRNAs) produced from transposons trigger silencing of those transposon loci; however, in this case, transposons escape host silencing by producing miRNA. Our analysis provides evidence for a novel mechanism by which transposons reduce host silencing, and it elucidates the front line of host genome–parasitic DNA interaction through the action of two types of small RNAs, siRNA and miRNA, produced from transposons.

## Introduction

RNA silencing is a mechanism mediated by small RNAs that regulates gene expression in eukaryotes at both the transcriptional and post-transcriptional levels. RNA silencing has a wide range of essential functions in cellular processes necessary for development of animals and plants, and it also has a role in defense against “genomic parasites” such as transposable elements (TEs) and viruses [1]–[3]. Silencing of TEs is triggered by small RNAs derived from the TE loci themselves. These small RNAs are usually 24 nt long in plants and are called small interfering RNAs (siRNAs). siRNAs are produced from TE transcripts by an enzyme called Dicer. Dicer acts on double-stranded RNA generated either by the action of RNA-dependent RNA polymerases or by transcription from both DNA strands. The TE-derived siRNAs are loaded onto Argonaute proteins, which degrade TE transcripts or repress translation by means of base-pairing between the transcripts and siRNAs [4]. In plants, TE-derived siRNAs also induce RNA-directed DNA methylation (RdDM), resulting in epigenetic inactivation of the TEs [5]–[7].

Although the majority of TEs are epigenetically silenced, most of the eukaryotic genome is composed of TEs and their remnants [8], [9]. This suggests that TEs have evolved countermeasures against host silencing [6], but the mechanisms by which TEs counteract silencing are poorly understood. In this paper, we demonstrate that a small RNA derived from certain TE loci suppresses the host silencing machinery. Generally, siRNAs produced from TEs trigger silencing of those same TEs; however, in this case, TEs escape host silencing by producing a class of miRNAs that acts on the host silencing machinery. Our analysis provides evidence for a novel mechanism by which transposons reduce host silencing, and it provides a glimpse of “front line” of host genome–parasitic DNA interactions through the action of small RNAs produced from the transposon.

## Results/Discussion

### Transposon-derived *miR820* targets *de novo* DNA methyltransferase gene *OsDRM2*

**Figure 1:**
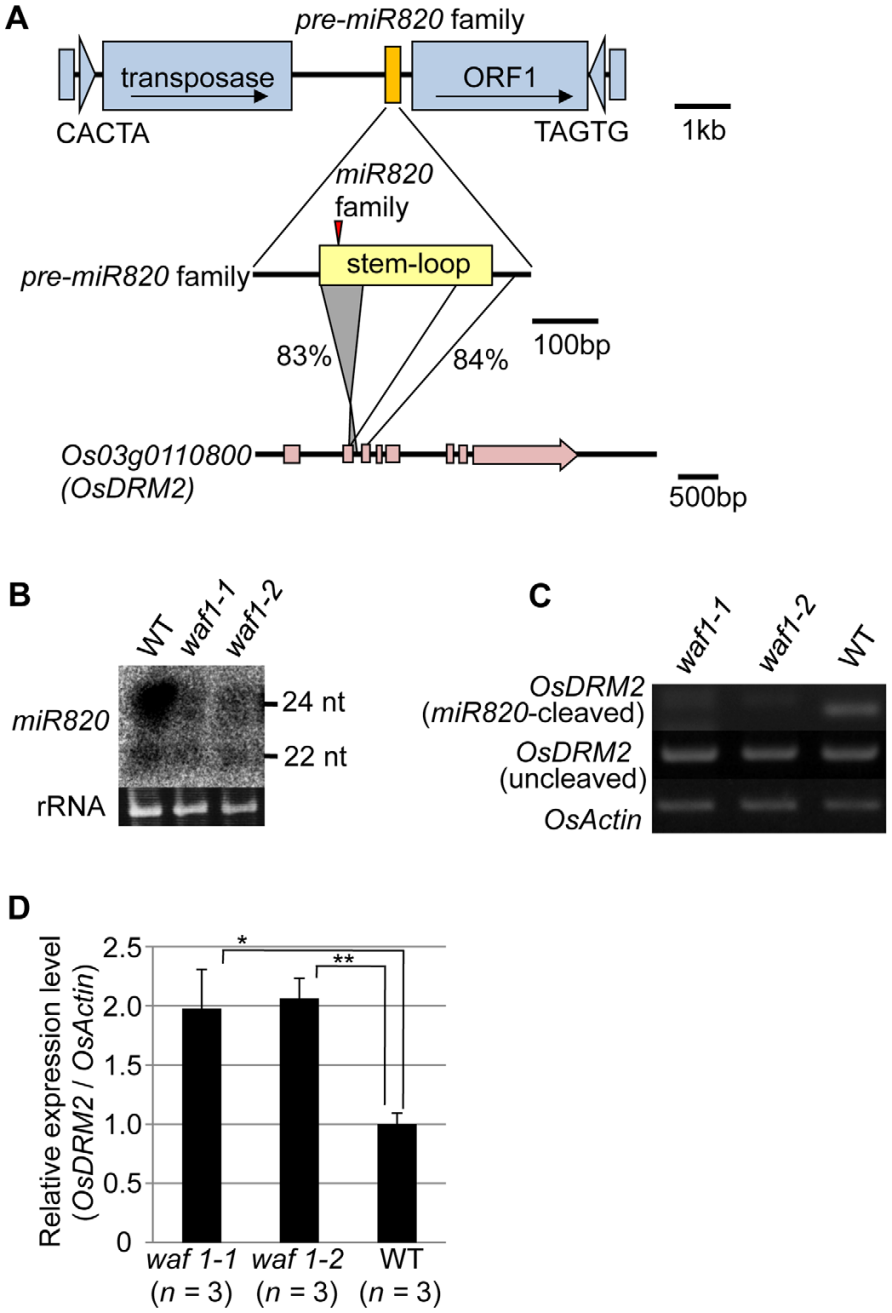
*miR820* family members are located within CACTA transposons and target the DNA methyltransferase gene *OsDRM2*. (A) The location of *miR820* within the CACTA DNA transposon is shown in the first row. The second and third rows indicate the similarity between the sequences of the *miR820* precursor (*pre-miR820*) and *Os03g0110800* (*OsDRM2*). The numbers beside the lines indicate the nucleotide identities between the regions. The red triangle indicates the location of *miR820* within the stem-loop region. (B) Northern blot analysis of *miR820* expression in the wild-type (WT) and in *waf1* mutants. (C) Detection of *miR820*-cleaved *OsDRM2* mRNA by RNA ligation–mediated 5′ RACE (upper panel). The same cDNA templates were used for PCR to amplify *OsDRM2* (middle panel) and *OsActin* (bottom panel) as controls for *OsDRM2* expression and RNA integrity. (D) qRT-PCR analysis measuring the expression level of *OsDRM2* in *waf1-1*, *waf1-2*, and WT. The expression level of the WT was set as 1. Values are means, with bars showing standard errors. Significance was assessed by a two-tailed Student’s t-test; (**) significant at the 1% level; (*) significant at the 5% level. The n value represents the number of mutant or wild-type individuals.

miRNAs are produced from stem structures formed within noncoding transcripts [10] and negatively regulate the expression of a range of plant genes, mainly by mRNA cleavage [11]. *miR820* is a small-RNA species with sizes of 22 and 24 nt [12], [13]. *miR820* is produced from transcripts originating from a region inside a class of CACTA DNA transposons in rice (Figure 1A). There are five copies of the CACTA transposon containing the *miR820* precursor (*pre-miR820*) in the rice (*Oryza sativa* L.) Nipponbare genome [14] (Figure S1A, S1B). Three of the *pre-miR820*s (*miR820a, -b, and –c*) encode the identical miRNA sequence [15], whereas *miR820d* and *miR820e* differ from the other three by one and two nucleotides, respectively (Figure S1C). The nucleotide sequences of the fold-back region of all five *pre-miR820* sequences show high sequence similarity to parts of *Os03g0110800* and the homologous region extends into the second exon and third intron of *Os03g0110800* (Figure 1A; Figures S2, S3). Thus, *pre-miR820* possibly originated from *Os03g0110800*, and the number of *pre-miR820* copies increases as the CACTA TEs propagate.

Because of this homology, *miR820* is predicted to target *Os03g0110800* (*OsDRM2*), which encodes a *de novo* DNA methyltransferase orthologous to *Arabidopsis DRM1/2* [15]–[19] (Figure S4A). It has been reported that the 24-nt species of *miR820* acts as a guide for DNA methylation at its target site, possibly through RdDM [13]. Indeed, we also confirmed the function of the 24-nt *miR820* species by detecting a high level of cytosine methylation specific to its presumed target site (Figure S4B). Because *pre-miR820* loci simultaneously produce both 22-nt and 24-nt miRNA species (Figure 1B), we investigated whether the 22-nt *miR820* species regulates *OsDRM2* expression through mRNA degradation by mapping the 22-nt *miR820* cleavage site of *OsDRM2*. We found a cleavage site at the predicted position for miRNA-based target gene cleavage (Figure S4C).

We further confirmed that this cleavage depends on the presence of *miR820* by using the *waf1* mutant in rice [20] (Figure 1B–1D). In *waf1*, accumulation of small RNAs is greatly decreased because of a mutation in *HEN1*, a gene encoding an RNA methyltransferase that is required for the stability of small RNAs [21]–[23]. In *waf1*, the expression levels of both the 22-nt and 24-nt species of *miR820* decreased compared to the wild-type (Figure 1B). To confirm that *OsDRM2* mRNA cleavage depends on the presence of *miR820*, we checked for the cleavage product in *waf1* mutants and in the wild-type. In the *waf1* mutants, there was no detectable cleavage of *OsDRM2* mRNA by *miR820* (Figure 1C). We also confirmed that the expression level of *OsDRM2* increased in *waf1* compared to the wild-type (Figure 1D). It is possible that this increase was not due solely to the loss of *miR820* because in *waf1*, the levels of most other small RNAs are also reduced [20]. However, considering that *OsDRM2* gave the highest hit score when *miR820* was used in BLAST searches against the entire rice genome (IRGSP Pseudomolecules 1.0) other than *miR820* itself, it is very likely that *miR820* negatively regulates the expression of *OsDRM2* at least in part.

### Negative regulation of *OsDRM2* by *miR820* activates TE expression

**Figure 2:**
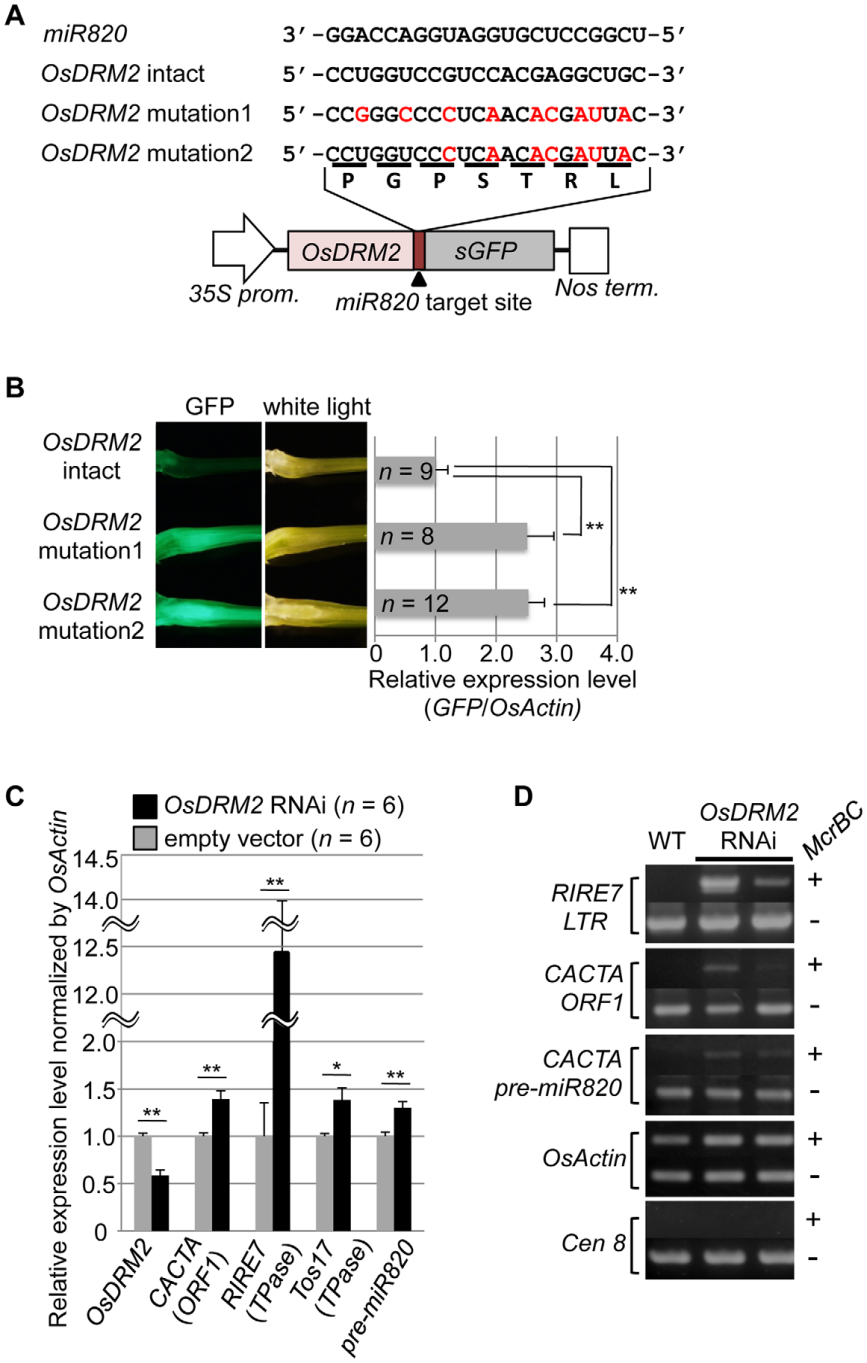
*OsDRM2* is negatively regulated by *miR820*. (A) The general structure of the *OsDRM2::GFP* fusion constructs is shown at the bottom, and the sequences of *miR820a/b/c* and the target sites in the *35S:OsDRM2 intact:GFP*, *35S:OsDRM2 mutation1:GFP*, and *35S:OsDRM2 mutation2:GFP* constructs are shown at the top. The changed nucleotides in the mutant genes are shown in red letters. (B) The panels on the left show GFP fluorescence and white-light observations of longitudinal sections of shoots of transgenic plants transformed with the constructs in (A). The graph at the right shows relative expression levels of *GFP* mRNA in the corresponding transgenic lines as measured by quantitative RT-PCR. The expression level of *OsDRM2*-intact lines was set as 1. (C) Relative expression levels of *OsDRM2* and TEs in *OsDRM2* RNAi transgenic callus (black bars) measured by qRT-PCR. The expression level of empty-vector lines was set as 1. (D) *McrBC*-PCR analysis of genomic DNA from callus of WT and two independent transgenic lines of *OsDRM2* RNAi. Two of the six *OsDRM2* RNAi transgenic lines analyzed in (C) were used. In (B) and (C), values are means, with bars showing standard errors. Significance was assessed by a two-tailed Student's *t*-test; (**) significant at the 1% level; (*) significant at the 5% level. The n value represents the number of independent transformants.

To test whether the expression level of *OsDRM2* depends on recognition by *miR820*, we made transgenic rice plants that express a fusion of a green fluorescent protein (GFP) gene and *OsDRM2* with or without synonymous mutations within the *miR820* recognition site; we then observed the GFP fluorescence and measured *GFP* mRNA levels (Figure 2A, 2B). As expected, the expression level of the *OsDRM2:GFP* fusion gene with an intact *miR820* recognition site was much lower than for those with synonymous mutations. In wild-type plants, both *miR820* and *OsDRM2* were expressed in all the tissues tested, although their expression levels differed between tissues (Figure S4D, S4G).

Next, we tested whether the expression patterns of *OsDRM2* and *miR820* overlapped. Northern analysis using total RNA extracted from vegetative shoots from two wild-type rice cultivars demonstrated that both genes were expressed within this tissue (Figure S4E). *In situ* hybridization experiments revealed that *OsDRM2* is ubiquitously expressed in vegetative shoots (Figure S4F). This suggests that the expression patterns of *miR820* and *OsDRM2* overlap at the cellular level, supporting the idea that *miR820* regulates *OsDRM2*. On the other hand, we did not observe a clear inverse relationship between the levels of *miR820* and *OsDRM2* expression. This might be because the expression levels of *miR820* and *OsDRM2* differed between tissues, and because *miR820* might reduce the amount of *OsDRM2* expression but not abolish it completely. Indeed, we found that overexpression of *pre-miR820* under the control of a strong constitutive promoter mildly reduced but did not eliminate the expression of *OsDRM2* (Figure S5A–S5D).

Because *de novo* DNA methyltransferase is a component of the host's silencing machinery [16]–[17], we tested whether reduced *OsDRM2* expression would affect the transcription of TEs by using transgenic rice plants in which *OsDRM2* expression was reduced by RNAi. We found that the expression levels of several TEs were increased in *DRM2* RNAi transgenic lines; furthermore, the expression levels of TEs such as *RIRE7* and CACTA carrying *pre-miR820* were inversely related to the degree of *DRM2* suppression (Figure 2C; Figure S6). Next, we observed the DNA methylation status at several TE loci by *McrBC*-PCR analysis (Figure 2D). In *OsDRM2* RNAi lines, DNA methylation within CACTA (including the *pre-miR820* region) and *RIRE7* is clearly reduced compared to the wild-type. Furthermore, we also observed elevated expression of *RIRE7* in the same *pre-miR820* overexpression experiment in which *OsDRM2* expression was found to be mildly reduced (Figure S5E). These experimental data are consistent with the idea that *OsDRM2* is involved in TE silencing through DNA methylation.

### The sequences of *miR820* and its target site in *OsDRM2* have co-evolved in BB/BBCC *Oryza* species

**Figure 3:**
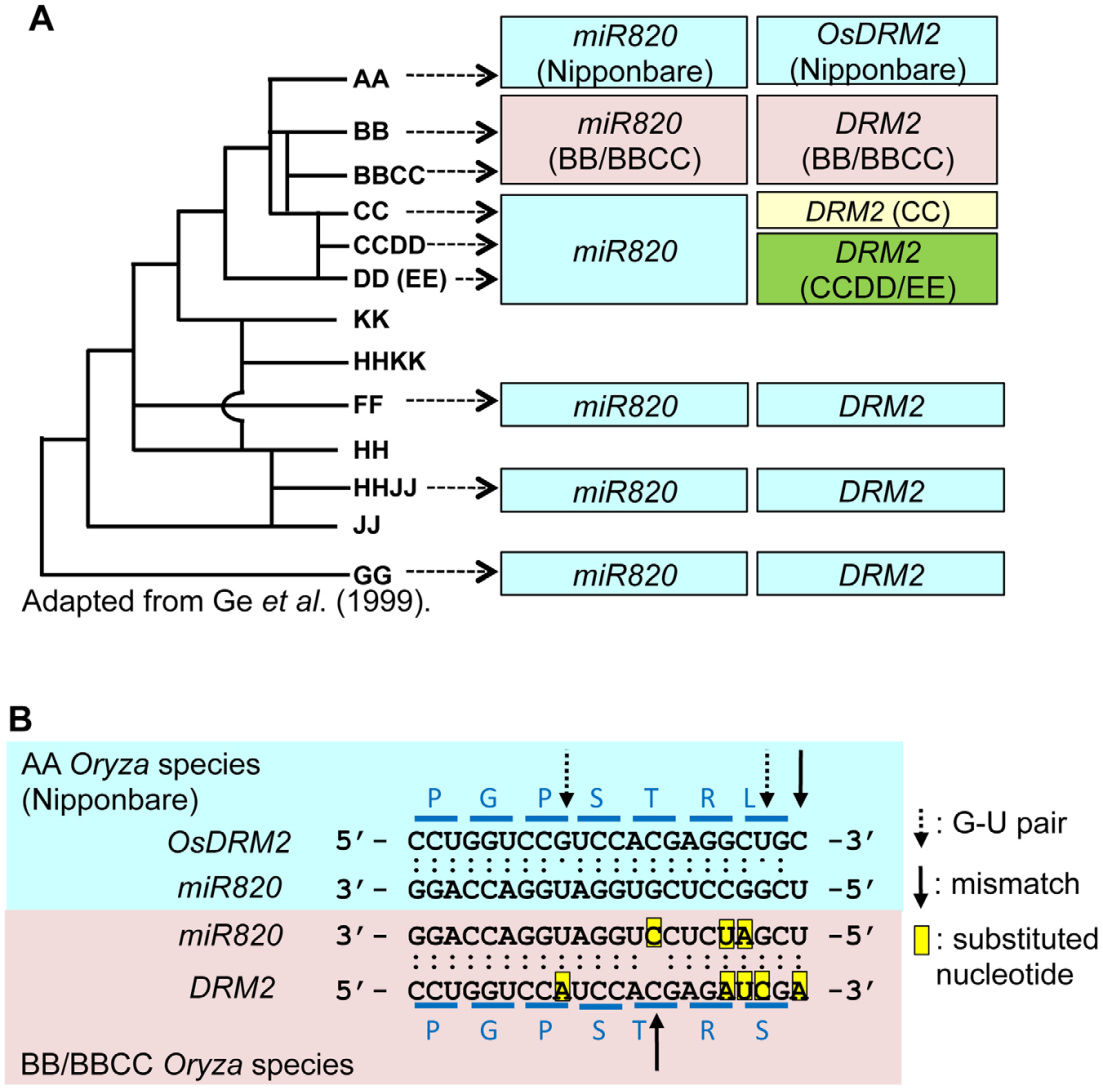
Regulation of *DRM2* by *miR820* is conserved among *Oryza* species. (A) The phylogenetic tree shows the evolutionary relationships between *Oryza* species (left). Boxes indicate the types of *miR820* sequences and *DRM2* target site sequences identified in each genome (right). Within each column, boxes of the same color indicate identical sequences. The genome origins of the sequences in the boxes are indicated by arrows. (B) Sequence alignments of *miR820* and its target site in *DRM2* in the AA (Nipponbare; blue box) and BB/BBCC genomes (pink box). Dots between nucleotides indicate the type of nucleotide pair: a double dot indicates an A-U or G-C pair, a single dot indicates a G-U pair, and no dot indicates a mismatch. The positions of G-U pairs and mismatches are shown with broken and solid arrows, respectively. The eight nucleotide substitutions found between AA and BB/BBCC *Oryza* species are highlighted in yellow. Blue lines above and below the sequence of *DRM2* indicate the codons. Letters above and below the lines indicate the amino acids encoded by *DRM2* in AA and BB/BBCC *Oryza* species, respectively. Phylogenetic tree in (A) adapted from Ge et al. (1999) [24].

We did not find *miR820* or its precursor sequence in the Arabidopsis or maize genome, suggesting that *miR820* is not widely conserved in plants. We then tested whether regulation by *miR820* is conserved among various *Oryza* species. We successfully amplified and sequenced both *miR820* and its recognition site in *DRM2* from the genomic DNAs of various accessions of *Oryza* [24] (Figure S7A, S7B; Table S1), strongly suggesting the conservation of this regulation mechanism among *Oryza* species. We recovered sequences identical to *miR820a/b/c* from all the *Oryza* genomes tested except for the BB and BBCC genomes (Figure S7A; Table S1). In species with BB or BBCC genomes, *miR820*-related sequences had three nucleotide substitutions compared with *miR820a/b/c*. Considering the phylogenetic relationships among *Oryza* species [24], the *miR820* sequence recovered from BB/BBCC *Oryza* species has diverged from *miR820a/b/c* (Figure 3A).

There are also several nucleotide substitutions in the *miR820* recognition site in *DRM2* in some *Oryza* genomes (Figure S7B). Remarkably, in the BB and BBCC genomes, there are five nucleotide substitutions in *DRM2*. Thus, in the BB and BBCC genomes, there are eight nucleotide substitutions in *miR820* and its recognition site in *DRM2*, compared with the corresponding *miR820* and target sequences in Nipponbare. This number of substitutions could greatly affect the capability of *miR820* to regulate *DRM2* in species with those genomes; however, the degree of base-pairing between *miR820* and its target site in *DRM2* in the BB and BBCC genomes is conserved (Figure 3B; Table S1). This indicates that, in BB/BBCC *Oryza* species, the sequences of *miR820* and its target site in *DRM2* have co-evolved to maintain the ability to form a stable RNA–RNA duplex. The co-evolution of these sequences strongly suggests that the regulation of *DRM2* by *miR820* is functional and that those nucleotide changes have accumulated as a result of the interplay between the host genome and the parasitic elements in these species.

### TEs carrying *pre-miR820* have proliferated in BB-genome species

**Figure 4:**
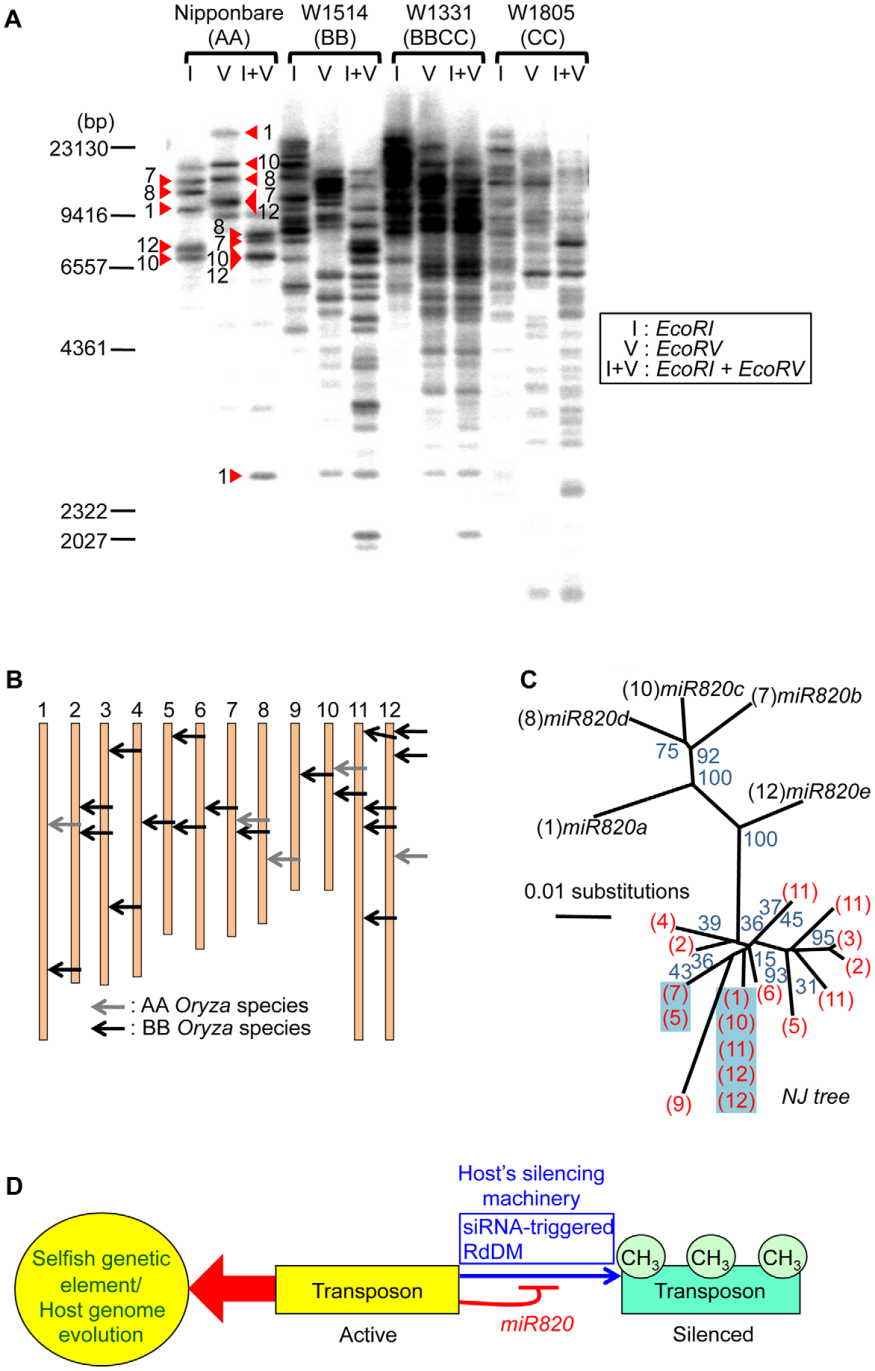
Increased copy number of CACTA carrying *pre-miR820* in the BB/BBCC genome. (A) Detection of CACTA TEs carrying *pre-miR820* by Southern blot analysis. Genomic DNA from AA, BB, BBCC, and CC *Oryza* species were digested with the enzymes indicated and probed with *pre-miR820*. Red triangles indicate the bands corresponding to the five copies of *pre-miR820* in Nipponbare. The number next to each triangle indicates the chromosome location of that copy. (B) Mapping of CACTA carrying *pre-miR820* in the rice genome. The genomic locations of CACTA carrying *pre-miR820* in AA and BB *Oryza* species are shown by gray arrows and black arrows, respectively. (C) Phylogenetic analysis of *miR820*-CACTA sequences. Bootstrap values (1000 replicates) are given for branch nodes. Black and red numbers in parentheses indicate the chromosome locations of *pre-miR820* sequences in the AA and BB genomes, respectively. Multiple copies on one branch, indicating that the identical sequence was found at multiple loci, are highlighted in blue. (D) A model for the regulation of *DRM2* by *miR820*. Active transposons behave as “selfish” genetic elements. This characteristic is counteracted by the host's silencing machinery (blue arrow), which acts to methylate and silence transposon loci. This action can be blocked by *miR820* (thin red line), which suppresses the host's silencing machinery and can drive host genome evolution (thick red arrow).

To see whether co-evolution of the nucleotide sequences of *DRM2* and *miR820* affected the behavior of TEs carrying *pre-miR820* in the BB genome, we performed Southern blot analysis to detect the copy number of CACTA carrying *pre-miR820* (Figure 4A). We found that the copy number of CACTA with *pre-miR820* was much higher in the BB/BBCC *Oryza* species than in the AA species Nipponbare. We also successfully determined the genomic locations of CACTA with *pre-miR820* in the BB genome (see Materials and Methods for details) and found that at least 18 copies of CACTA with *pre-miR820* are dispersed throughout this genome (Figure 4B). We also sequenced the CACTA with *pre-miR820* in BB-genome species and conducted phylogenetic analysis using *pre-miR820* sequences from Nipponbare and BB-genome species. This analysis revealed a sudden increase in copy number of CACTA carrying *pre-miR820*, in which identical sequences around the *pre-miR820* region were recovered from multiple loci (Figure 4C). Because the *miR820* sequence in the BB species shown in Figure 3B was obtained by direct sequencing of PCR products, it should be representative of the *miR820* sequence in BB species. In fact, the majority (11 out of 18 copies) of *pre-miR820* found in the BB genome carries the same *miR820* sequence as the one recovered by direct sequencing, which is also the sequence that would form the most stable hybrid with the *DRM2* sequence found in the BB genome. These results suggest that the CACTA transposon with this *miR820* sequence was predominantly proliferated or maintained, and became the predominant *miR820* in BB species.

We hypothesize the following scenario as a mechanism connecting the co-evolution of *miR820* and *DRM2* and the rapid increase in the copy number of CACTA carrying *pre-miR820*. When *OsDRM2* expression decreases, possibly because of nucleotide substitutions within *miR820* that enable it to form more stable hybrids with *OsDRM2* or for other reasons, more *miR820* can be produced, possibly because host-mediated silencing is suppressed efficiently. Indeed, RNAi-mediated suppression of *OsDRM2* increased *pre-miR820* expression (Figure S6C). This is expected to drive the selection of nucleotide substitutions at *miR820* or at its target site because drastic reduction of *OsDRM2* levels could be lethal. This hypothesis is supported by the fact that we recovered *OsDRM2* RNAi transgenic plants with about half the normal expression level of *OsDRM2* (Figure 2C). Thus, there should be selection pressure for mutations within the miRNA target site of *OsDRM2*. In turn, TE would favor changes in the *miR820* sequence that correspond to the changes in the target site. This evolutionary “arms race”, in which hosts and parasitic DNA co-evolve, allows nucleotide substitutions to accumulate within both the miRNA and its target sequence, which maintains the ability to form stable hybrids between them. This might account for the fact that, in BB species, the most predominant CACTAs with *pre-miR820* were those that could form the most stable hybrid with the target sequence.

A model for the regulation of *DRM2* by *miR820* sequences is shown in Figure 4D. In general, TE-derived small RNAs act as a trigger for silencing [5]–[7]. However, in this case, transposons that incorporate miRNA genes that target the host's silencing machinery are able to counteract the host's defense system. Similar examples of “arms races” between hosts and parasites are well documented in studies of plant RNA viruses and their hosts [25], in which RNA viruses that encode silencing-suppressor proteins are able to escape silencing by the host. Our model for the regulation of *DRM2* by *miR820* predicts that this regulation might affect not only CACTA carrying *pre-miR820* but also other TEs. Indeed, in *DRM2* RNAi lines, we observed upregulation of expression from TEs other than CACTA (Figure 2C). However, considering that BB-containing species have relatively small genomes compared with other *Oryza* species [26], the downregulation of *DRM2* by *miR820* would not be expected to affect a large number of TEs in BB-containing species. Rather the effect might be relatively specific to particular TEs or their lineage, as has been observed for the *Arabidopsis ddm1* mutation [27].

So far, *miR820* has been found only in rice, suggesting a recent origin. The primary and secondary structures of *pre-miR820* also support this idea, because *pre-miR820* still shows high homology within its stem parts to the intron sequence of *OsDRM2*. In general, non-conserved miRNA genes evolve very fast and they often appear and disappear from the genome. Considering that *miR820* is encoded by parasitic DNA and its primary function seems to be as an anti-host agent, it is possible that *miR820* might be lost in the future, as is often the case for non-conserved miRNA genes. However, it is intriguing to speculate that *miR820* might function not only as an anti-host mechanism for parasites but also in a way that is beneficial for the host. The co-evolution of *miR820* and its recognition site in BB/BBCC species supports this idea. It is possible that, in order to adapt against genomic stresses such as climate or environmental changes, the host maintained or created genome flexibility by keeping or allowing *DRM2* under the regulation of *miR820* in BB species in the past. Thus, our analysis of the regulation of *DRM2* by *miR820* sheds light on the action of two types of transposon-derived small RNAs, siRNA and miRNA, in the battles and possibly even the cooperation between plant genomes and their parasites.

## Materials and Methods

### Plant materials

Wild-type Nipponbare and *waf1* mutant rice plants were grown in soil or in tissue culture boxes at 29°C under continuous light. DNA, plants, and seeds of *Oryza* species were kindly provided by the National Institute of Genetics (Mishima, Japan).

### Plasmid construction and production of transgenic plants

*OsDRM2* cDNA was kindly provided by Dr. S. Iida, Shizuoka Prefectural University (Shizuoka, Japan). The *p35S:OsDRM2 intact:GFP*, *p35S:OsDRM2 mutation1:GFP*, and *p35S:OsDRM2 mutation2:GFP* vectors were constructed by introducing mutations using the GeneTailor Site-Directed Mutagenesis System (Invitrogen). Next, the part of each *OsDRM2* cDNA that included the *miR820* target site was amplified and cloned into the pENTR/D-TOPO vector (Invitrogen). The resultant vectors containing the cDNA fragments were introduced into the pGWB5 binary vector [28], which carries a GFP reporter gene driven by the 35S promoter, by using Gateway technology (Invitrogen). For *pAct:pre-miR820:Nos* construction, a 0.5-kb *pre-miR820* fragment was amplified and inserted into the pCRII vector (Invitrogen). A *pre-miR820* fragment was then excised with *Xba*I and *Sma*I, and cloned into the binary vector carrying the rice *Actin* gene promoter and *Nos* terminator. For *pAct:OsDRM2 RNAi:Nos* construction, a 0.9-kb *OsDRM2* cDNA fragment with *Pst*I and *Xba*I linkers was cloned into the *Pst*I and *Xba*I sites of the pBS-SK vector containing a partial *GUS* fragment at its *EcoR*V site. Similarly, a cDNA fragment with *Hind*III and *Sma*I/*Apa*I linkers was inserted into the *Hind*III and *Apa*I sites of the vector. The resultant vector was cloned into the *Xba*I and *Sma*I sites of a binary vector carrying the rice *Actin* gene promoter and *Nos* terminator. These binary vectors were introduced into *Agrobacterium* strain EHA101 and used for transformation of rice by the standard method [29]. The primers used for vector construction are listed in Table S2.

### RNA analysis

Total RNA was isolated from shoots of *waf1* and various tissues of Nipponbare wild-type non-transgenic plants; shoots of *p35S:OsDRM2 intact:GFP*, *p35S:OsDRM2 mutation1:GFP*, and *p35S:OsDRM2 mutation2:GFP* T_2_ plants; and calli of *pAct:pre-miR820* and *pAct:OsDRM2 RNAi* by using TRIzol reagent (Invitrogen). For analysis of *waf1* and wild-type plants and of *pAct:pre-miR820:Nos* and *pAct:OsDRM2 RNAi:Nos* callus, 10 µg of each RNA sample was loaded onto an agarose or acrylamide gel (for analysis of *OsDRM2* and *miR820a/b/c*, respectively), separated by electrophoresis, and blotted onto nylon membranes. The membranes were probed with oligo DNA complementary to *miR820a/b/c* or *OsDRM2* cDNA, depending on the experiment.

### 5′ RACE

Total RNA was purified with the RNeasy Mini Kit (QIAGEN) according to the manufacturer's instructions. 3 µg of purified total RNA was subjected to RNA Oligo ligation with the GeneRacer Kit (Invitrogen) according to the manufacturer's instructions. The oligo-ligated RNA was reverse-transcribed using Omniscript Reverse Transcriptase (QIAGEN) with random primers (N_9_). PCR and nested PCR were performed using *Ex Taq* DNA polymerase (TaKaRa). Primers used for 5′ RACE PCR are listed in Table S2. Amplified bands were gel-purified, cloned, and sequenced.

### RT–PCR

Relative expression levels were quantified using the StepOnePlus Real-Time PCR system (Applied Biosystems) and the One Step SYBR PrimeScript RT-PCR Kit II (TaKaRa). The quantitative RT-PCR reactions contained 5 µl 2× One Step SYBR RT-PCR Buffer 4, 0.5 µl DMSO, 0.4 µl PrimeScript 1 step Enzyme Mix 2, 0.2 µl 50× ROX reference dye, 50 ng total RNA, and 400 nM of each primer, and were run in triplicate. The mixtures were first reverse-transcribed at 42°C for 5 min, then amplified via PCR using a two-step cycling program (95°C for 5 s, 60°C for 20 s) for 40 cycles. Quantitative RT-PCR specificity was checked for each run with a dissociation curve, at temperatures ranging from 95°C to 60°C. Data from quantitative RT-PCR were analyzed using the standard-curve method. The housekeeping genes *OsActin* and *OsGAPDH* were used to normalize the quantitative RT-PCR output. Primers used for quantitative RT-PCR are listed in Table S2.

### *McrBC*–PCR

Genomic DNAs were isolated from wild-type nontransgenic and *pAct:OsDRM2 RNAi:Nos* calli. For *McrBC*-PCR analysis, 500 ng of genomic DNAs were digested with or without 40 units of *McrBC* restriction enzyme (New England Biolabs) for 12 hr. PCR was performed using *Ex Taq* DNA polymerase (TaKaRa). Primers used for PCR are listed in Table S2. *OsActin* and *Centromere 8* are controls for regions with low and high DNA methylation, respectively.

### Sequence analysis

Genomic DNA samples from various *Oryza* species were kindly provided by the National Institute of Genetics (Mishima, Japan). We amplified both *miR820* and its target site in *DRM2* by PCR using the primers listed in Table S2. The amplified DNA fragments were gel-purified and used as templates for direct sequencing. The miRNA target score was calculated for each *miR820*:*DRM2* duplex based on the method described in [30]. To detect the copy number of CACTA TEs carrying *miR820* by Southern blot analysis, genomic DNA samples were extracted from leaves of Nipponbare (AA), W1514 (BB), W1331 (BBCC), and W1805 (CC), treated with RNase A, and digested with restriction enzymes. These samples were loaded onto an agarose gel, separated by electrophoresis, blotted onto a nylon membrane, and probed with the *pre-miR820* DNA fragment.

### Mapping of CACTA carrying *pre-miR820*

Our strategy to map *miR820*-CACTA from BB-genome species was based on the synteny between AA and BB *Oryza* species [26]. Briefly, by screening the BAC library of a BB-genome species, we identified BAC clones carrying *miR820*-CACTA from BB. Then, using the BAC end sequences of these clones deposited to database, we identified the corresponding physical position of these clones in the Nipponbare genome. This strategy is advantageous over other methods, such as transposon display, to monitor the varieties of transposon, especially long transposons with specific internal sequences, because transposon display identifies only the ends of transposon sequences. The precise method used for this experiment was as follows: A BAC filter and library of *Oryza punctata* (genome BB) genomic DNA were purchased from the Arizona Genomics Institute (Tucson, AZ). By screening these libraries using a labeled *pre-miR820* DNA fragment, we identified 48 BAC clones carrying *miR820*-CACTA. We confirmed that these clones carried *miR820*-CACTA by PCR amplification and sequencing of the region around *pre*-*miR820* in CACTA. Using the BAC end sequence obtained from http://www.omap.org/, we located those BACs on a physical map of the Nipponbare rice genome. Multiple sequence alignment for the phylogenetic analysis was constructed using Clustal X, and an unrooted tree was made by the neighbor-joining method [31] using PAUP 4.0 software (Sinauer Associates).

### *In situ* mRNA hybridization

In situ hybridization was performed as previously described by Kouchi and Hata (1993) [32]. For the OsDRM2 probe, the full-length cDNA clone was used as a template for in vitro transcription. Hybridizations were conducted at 55°C overnight; slides were then washed four times at 50°C for 10 min each. An excess amount of sense transcript was used as negative control.

## Supporting Information

Figure S1 Structures and sequences of the five copies of *miR820* in Nipponbare rice. (A) Schematic representations of the structures of the five copies of CACTA transposons carrying *miR820*. (B) Stem-loop structures of the five copies of *pre-miR820* in Nipponbare, predicted by the mfold program. The *miR820* sequences are designated by red rectangles. (C) Sequence alignment of members of the *miR820* family, *miR820a–e*, in Nipponbare rice. Dots indicate identical nucleotides.
(TIF)

Figure S2 Multiple sequence alignment of the five copies of *pre-miR820* in the Nipponbare genome. Sequence alignment was made using ClustalX at default settings. The blue lines, red box, and black box indicate the regions corresponding to the stem-loop structure, *miR820*, and miR820*, respectively.
(TIF)

Figure S3 Multiple sequence alignment of the five copies of *pre-miR820* and a part of the *OsDRM2* sequence in the Nipponbare genome. Sequence alignment was made using ClustalX at default settings. The blue line, black lines, and red box indicate the regions corresponding to the *OsDRM2* second exon, third intron, and miR820*, respectively, in the *pre-miR820* sequences and the *miR820* recognition site in *OsDRM2*.
(TIF)

Figure S4 The target of *miR820* is *OsDRM2*. (A) Phylogenetic tree of *de novo* DNA methyltransferases. Amino acid sequences in the Cyt-C5 DNA methylase domain were used for this analysis. The bootstrap values from 1000 replicates are indicated at each node. Os, rice; Z and Zm, maize; At, Arabidopsis; Nt, tobacco; Mt, Medicago; Hv, barley; Hs, human. (B) Analysis of DNA methylation status of *OsDRM2* in wild-type rice by bisulfite sequencing. The colored vertical lines above and below the bold black bar show the percentage of methylation and the position of individual cytosine sites, respectively. The target site of *miR820* is indicated by the red bar. (C) Mapping of the *miR820* cleavage site in *OsDRM2*. The arrow indicates the position of the cleaved end. The numbers above the arrow denote the number of clones ending at this position (left) and the total number of clones sequenced (right). (D) Northern blot analysis of *miR820* expression in various tissues. (E) Northern blot analysis of *OsDRM2* expression in various tissues. (F) Northern blot analysis of *miR820* and *OsDRM2* expression in vegetative shoots of two wild-type (WT) strains, Nipponbare and T-65. (G) *In situ* mRNA localization of *OsDRM2* in the vegetative shoot of Nipponbare using anti-sense probe (left panel) and the excess amount of sense probe as control (right panel), respectively. Bars = 50 um.
(TIF)

Figure S5 Overexpression of *pre-miR820* decreases *OsDRM2* expression. (A) Relative expression levels of *pre-miR820* measured by qRT-PCR in *pre-miR820* overexpression lines (n = 5) and empty-vector lines (n = 6). The expression level of *pre-miR820* was normalized to *OsGAPDH* and the expression level of the empty-vector lines was set as 1. (B) Northern blot analysis showing increased *miR820* expression in two independent transgenic lines transformed with the *pre-miR820* overexpression construct compared to empty-vector controls. (C) Northern blot analysis showing decreased *OsDRM2* expression in two *pre-miR820* overexpression lines compared to empty-vector controls; line numbers correspond to those in (B). (D) Relative expression levels of *OsDRM2* measured by qRT-PCR in the same transgenic lines as in (A). The expression level of *OsDRM2* was normalized to *OsGAPDH*. The expression level of empty-vector lines was set as 1. (E) Relative expression levels of *RIRE7* measured by qRT-PCR in the same transgenic lines as in (C). The expression level of *RIRE7* was normalized to *OsGAPDH*. The expression level of empty-vector lines was set as 1. In (A), (D) and (E), values are means, with bars showing standard errors. In (A) and (D), significance was assessed by a two-tailed Student's *t*-test; (*) significant at the 5% level.
(TIF)

Figure S6 Decreased expression of *OsDRM2* by RNAi is associated with increased TE expression. (A–C) Relative expression levels of *OsDRM2* (A), *RIRE7* (B), and the *pre-miR820* region of CACTA (C) in independent transgenic lines transformed with an *OsDRM2* RNAi construct (n = 6) or an empty vector (n = 3). Relative expression levels were measured by qRT-PCR and normalized to *OsActin*. Data shown are means of three technical replicates, with bars representing the standard errors. The relative expression level of *OsDRM2* RNAi #1 was set to 1.
(TIF)

Figure S7 Sequence alignments of *miR820* and its target site in *DRM2* among *Oryza* species. (A) Alignment of sequences of *miR820* among various *Oryza* species. (B) Alignment of sequences of the *miR820* target site in *DRM2* among various *Oryza* species. Dots indicate nucleotides identical to those in Nipponbare *miR820a/b/c* (A) or *DRM2* (B).
(TIF)

Table S1 Sequencing analysis of *miR820* and its target site in *DRM2* among various *Oryza* species.
(DOCX)

Table S2 Primers used in this study.
(DOCX)
