# Supplementary figures and images for: Role of Transposon-Derived Small RNAs in the Interplay between Genomes and Parasitic DNA in Rice
Source: PLoS Genet. 2012 Sep 27;8(9):e1002953. doi: 10.1371/journal.pgen.1002953 (PMC3459959; doi:10.1371/journal.pgen.1002953)

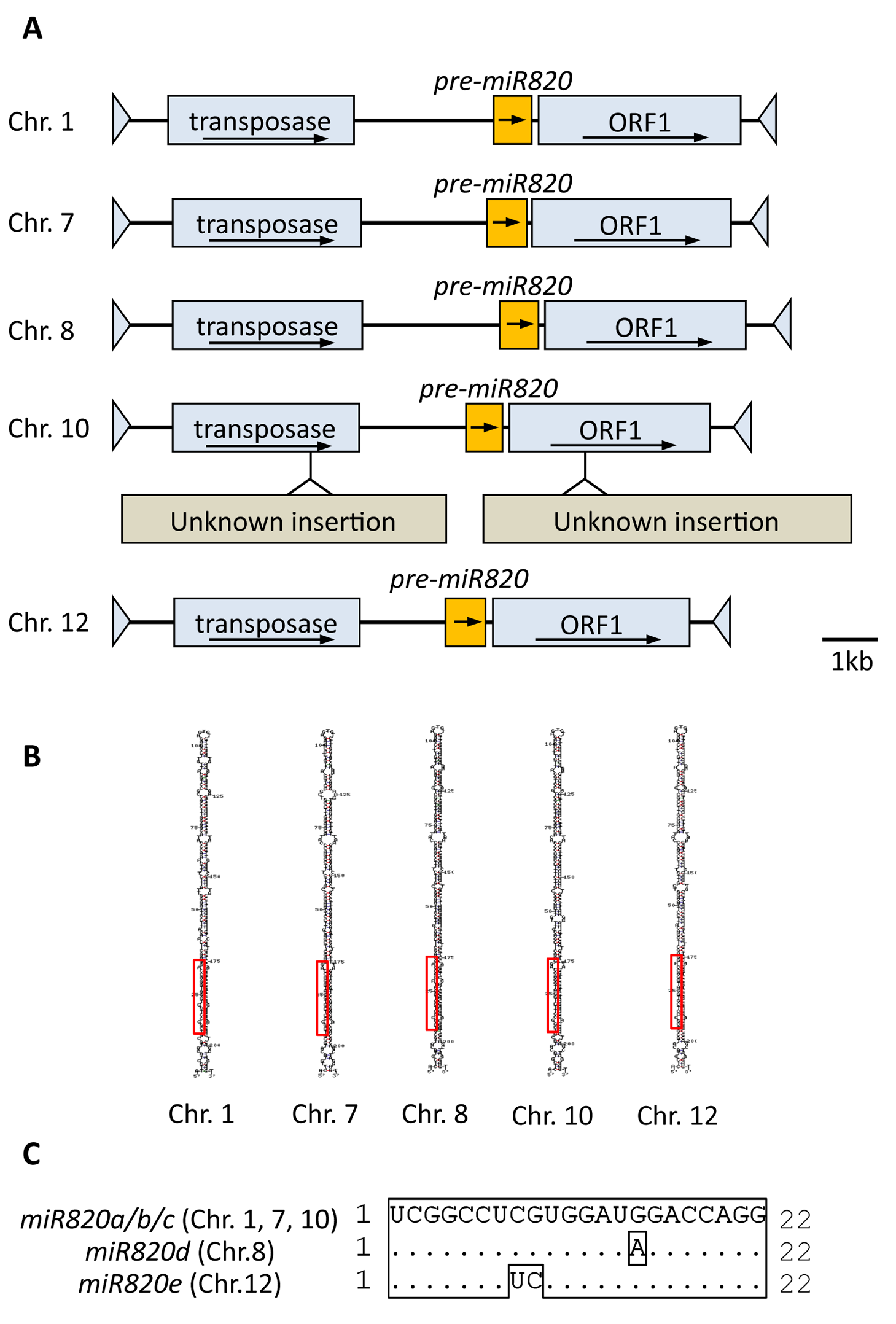

Supplement: Figure S1 — Structures and sequences of the five copies of miR820 in Nipponbare rice. (A) Schematic representations of the structures of the five copies of CACTA transposons carrying miR820. (B) Stem-loop structures of the five copies of pre-miR820 in Nipponbare, predicted by the mfold program. The miR820 sequences are designated by red rectangles. (C) Sequence alignment of members of the miR820 family, miR820a–e, in Nipponbare rice. Dots indicate identical nucleotides. (TIF) [file pgen.1002953.s001.tif]

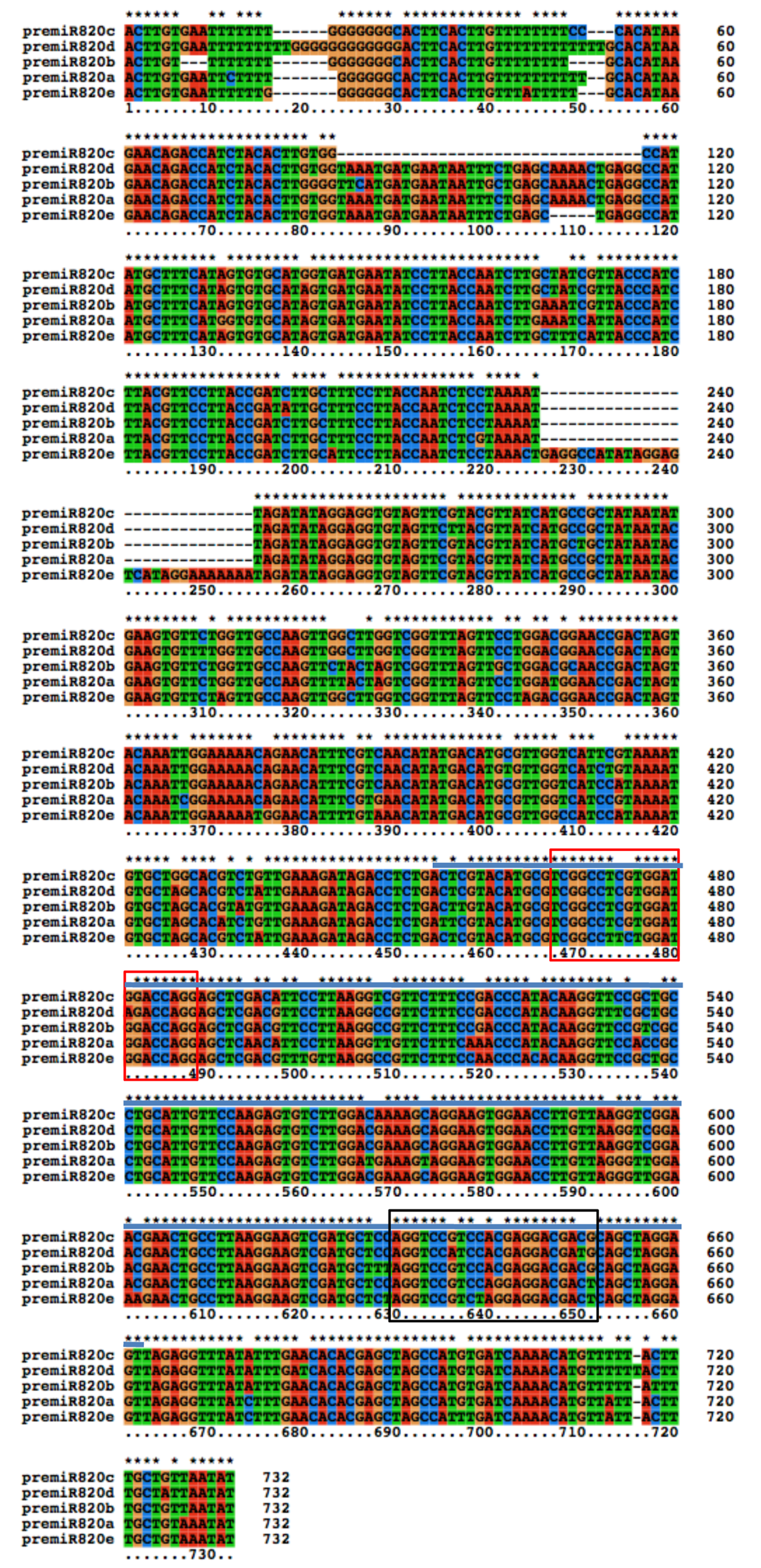

Supplement: Figure S2 — Multiple sequence alignment of the five copies of pre-miR820 in the Nipponbare genome. Sequence alignment was made using ClustalX at default settings. The blue lines, red box, and black box indicate the regions corresponding to the stem-loop structure, miR820, and miR820*, respectively. (TIF) [file pgen.1002953.s002.tif]

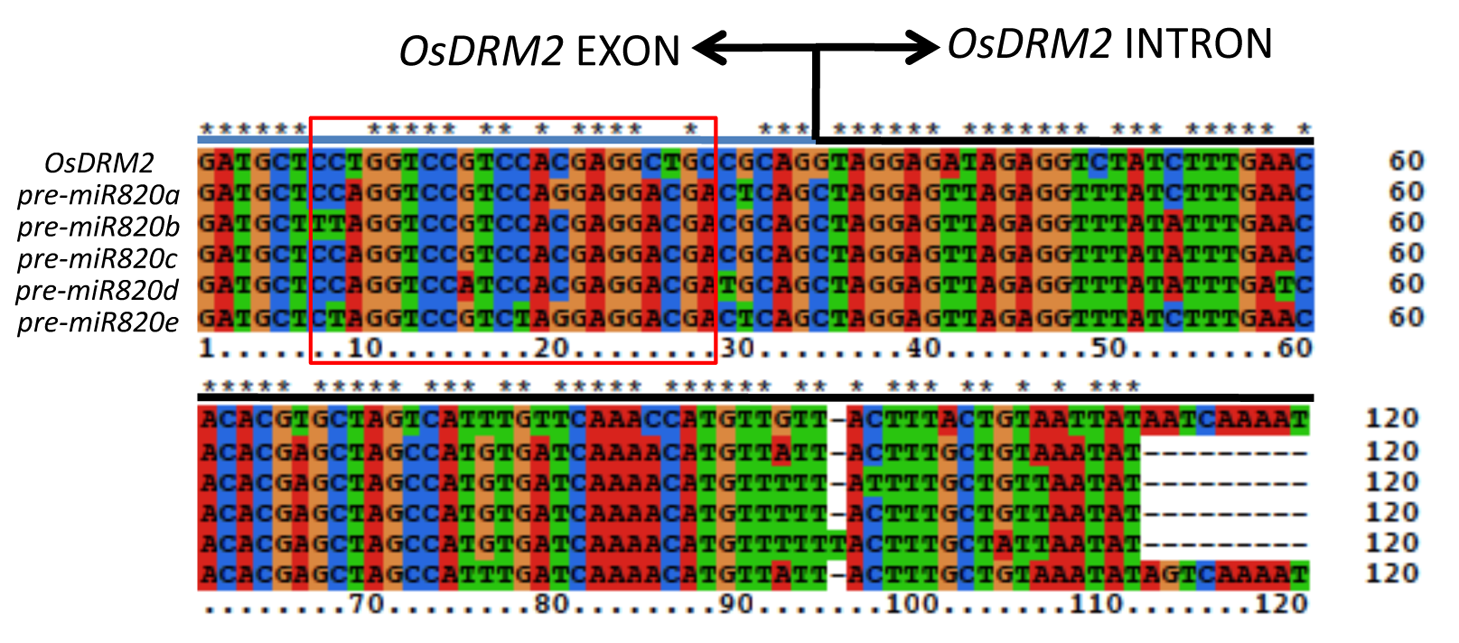

Supplement: Figure S3 — Multiple sequence alignment of the five copies of pre-miR820 and a part of the OsDRM2 sequence in the Nipponbare genome. Sequence alignment was made using ClustalX at default settings. The blue line, black lines, and red box indicate the regions corresponding to the OsDRM2 second exon, third intron, and miR820*, respectively, in the pre-miR820 sequences and the miR820 recognition site in OsDRM2. (TIF) [file pgen.1002953.s003.tif]

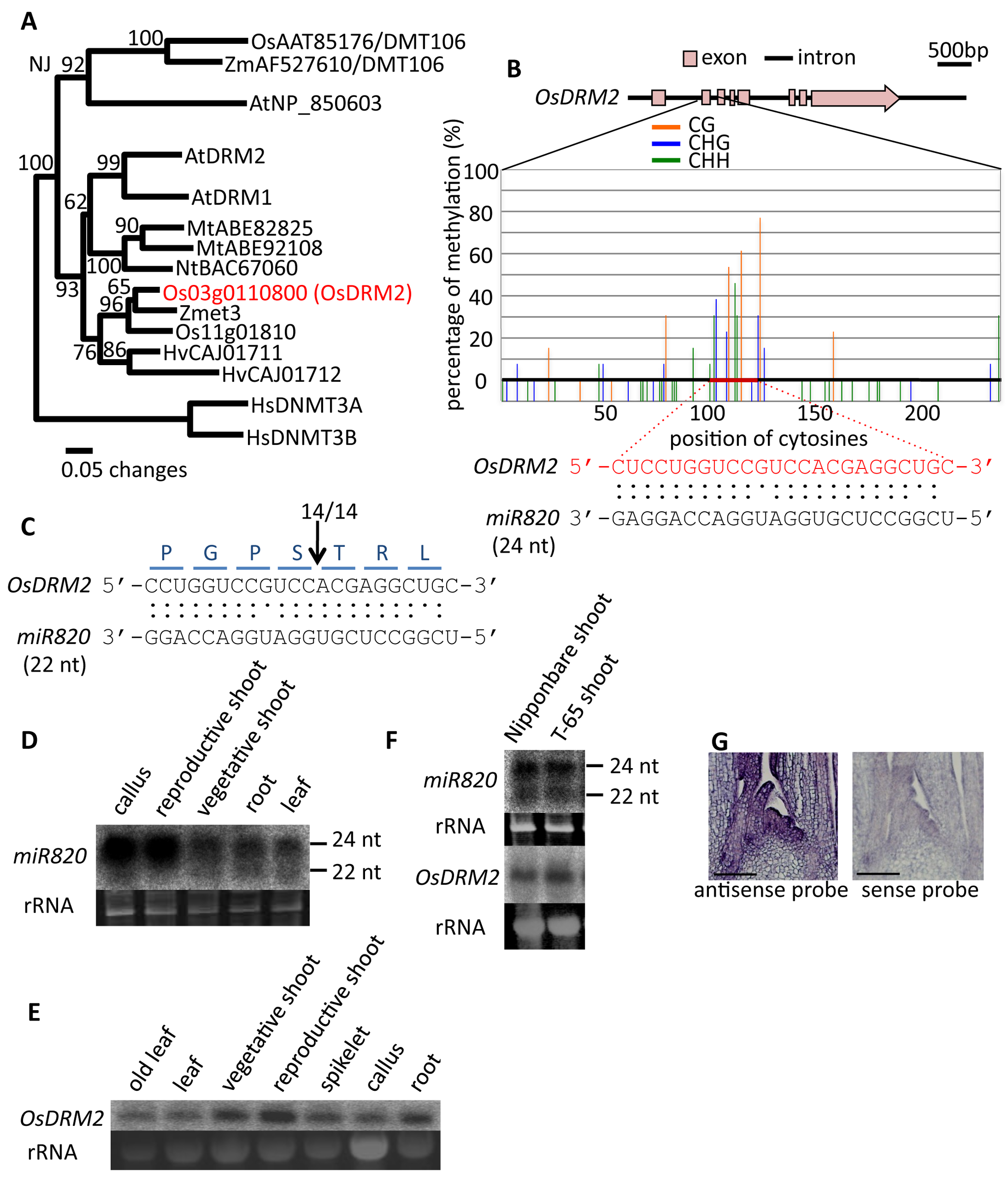

Supplement: Figure S4 — The target of miR820 is OsDRM2. (A) Phylogenetic tree of de novo DNA methyltransferases. Amino acid sequences in the Cyt-C5 DNA methylase domain were used for this analysis. The bootstrap values from 1000 replicates are indicated at each node. Os, rice; Z and Zm, maize; At, Arabidopsis; Nt, tobacco; Mt, Medicago; Hv, barley; Hs, human. (B) Analysis of DNA methylation status of OsDRM2 in wild-type rice by bisulfite sequencing. The colored vertical lines above and below the bold black bar show the percentage of methylation and the position of individual cytosine sites, respectively. The target site of miR820 is indicated by the red bar. (C) Mapping of the miR820 cleavage site in OsDRM2. The arrow indicates the position of the cleaved end. The numbers above the arrow denote the number of clones ending at this position (left) and the total number of clones sequenced (right). (D) Northern blot analysis of miR820 expression in various tissues. (E) Northern blot analysis of OsDRM2 expression in various tissues. (F) Northern blot analysis of miR820 and OsDRM2 expression in vegetative shoots of two wild-type (WT) strains, Nipponbare and T-65. (G) In situ mRNA localization of OsDRM2 in the vegetative shoot of Nipponbare using anti-sense probe (left panel) and the excess amount of sense probe as control (right panel), respectively. Bars = 50 um. (TIF) [file pgen.1002953.s004.tif]

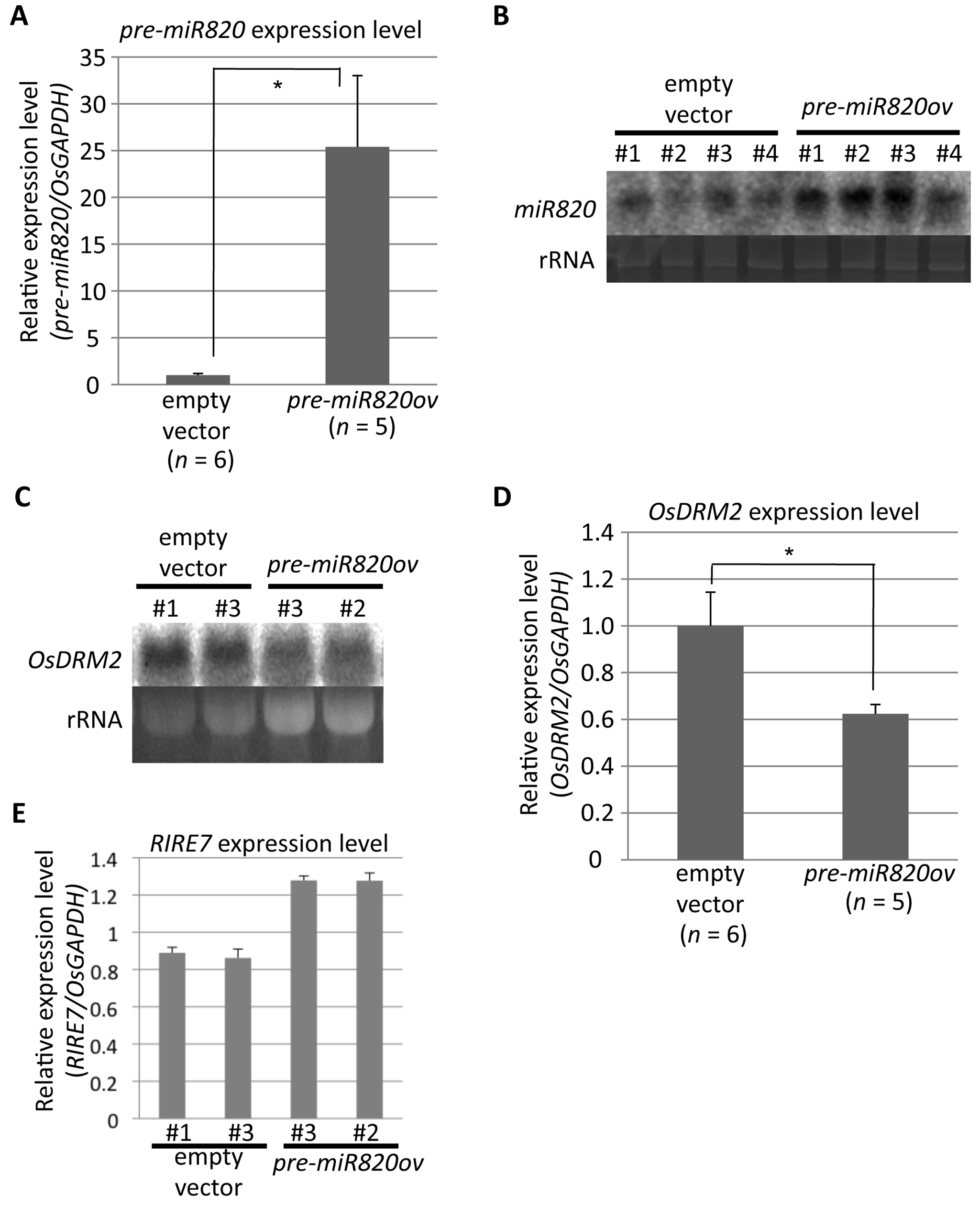

Supplement: Figure S5 — Overexpression of pre-miR820 decreases OsDRM2 expression. (A) Relative expression levels of pre-miR820 measured by qRT-PCR in pre-miR820 overexpression lines (n = 5) and empty-vector lines (n = 6). The expression level of pre-miR820 was normalized to OsGAPDH and the expression level of the empty-vector lines was set as 1. (B) Northern blot analysis showing increased miR820 expression in two independent transgenic lines transformed with the pre-miR820 overexpression construct compared to empty-vector controls. (C) Northern blot analysis showing decreased OsDRM2 expression in two pre-miR820 overexpression lines compared to empty-vector controls; line numbers correspond to those in (B). (D) Relative expression levels of OsDRM2 measured by qRT-PCR in the same transgenic lines as in (A). The expression level of OsDRM2 was normalized to OsGAPDH. The expression level of empty-vector lines was set as 1. (E) Relative expression levels of RIRE7 measured by qRT-PCR in the same transgenic lines as in (C). The expression level of RIRE7 was normalized to OsGAPDH. The expression level of empty-vector lines was set as 1. In (A), (D) and (E), values are means, with bars showing standard errors. In (A) and (D), significance was assessed by a two-tailed Student's t-test; (*) significant at the 5% level. (TIF) [file pgen.1002953.s005.tif]

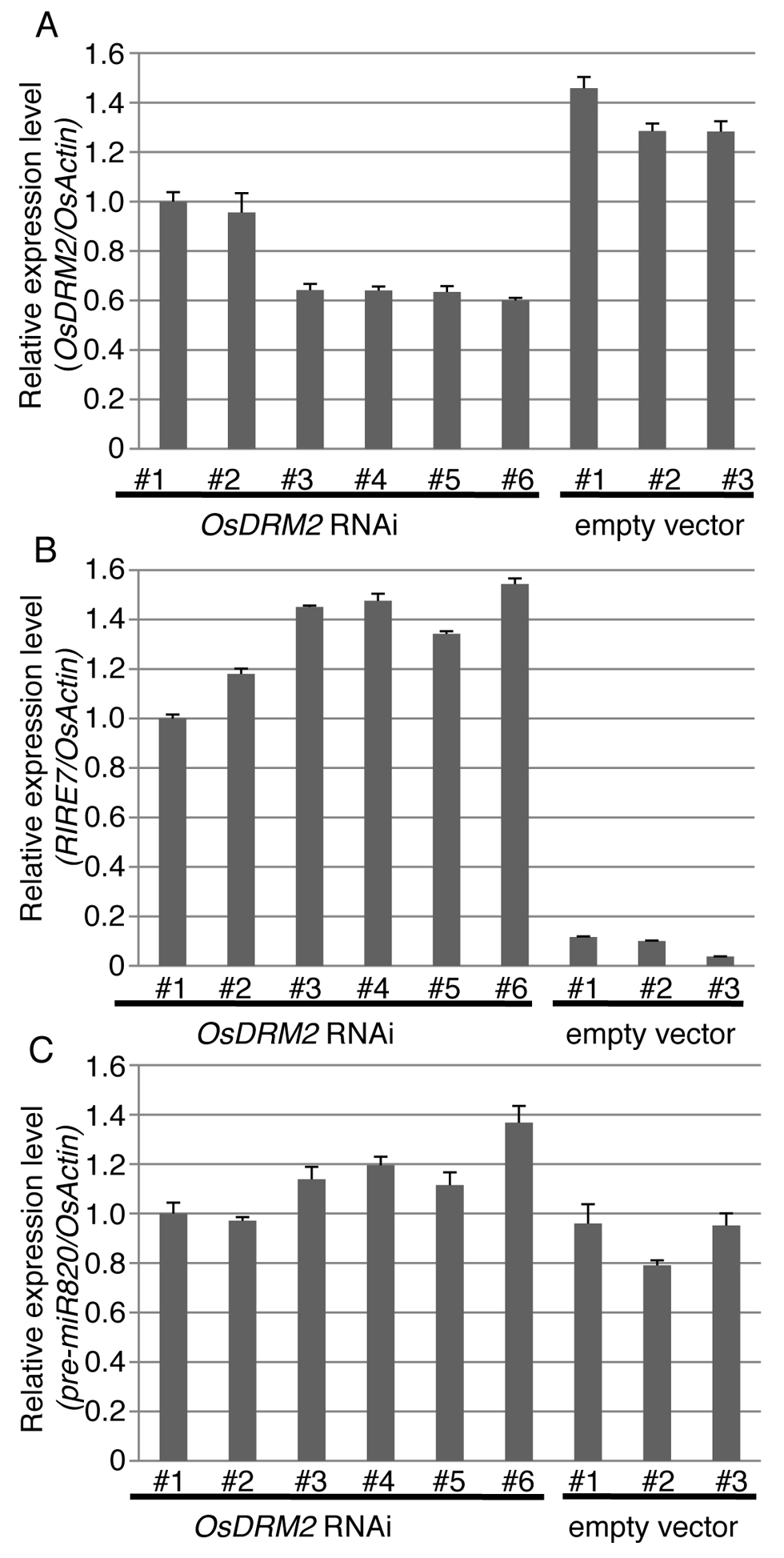

Supplement: Figure S6 — Decreased expression of OsDRM2 by RNAi is associated with increased TE expression. (A–C) Relative expression levels of OsDRM2 (A), RIRE7 (B), and the pre-miR820 region of CACTA (C) in independent transgenic lines transformed with an OsDRM2 RNAi construct (n = 6) or an empty vector (n = 3). Relative expression levels were measured by qRT-PCR and normalized to OsActin. Data shown are means of three technical replicates, with bars representing the standard errors. The relative expression level of OsDRM2 RNAi #1 was set to 1. (TIF) [file pgen.1002953.s006.tif]

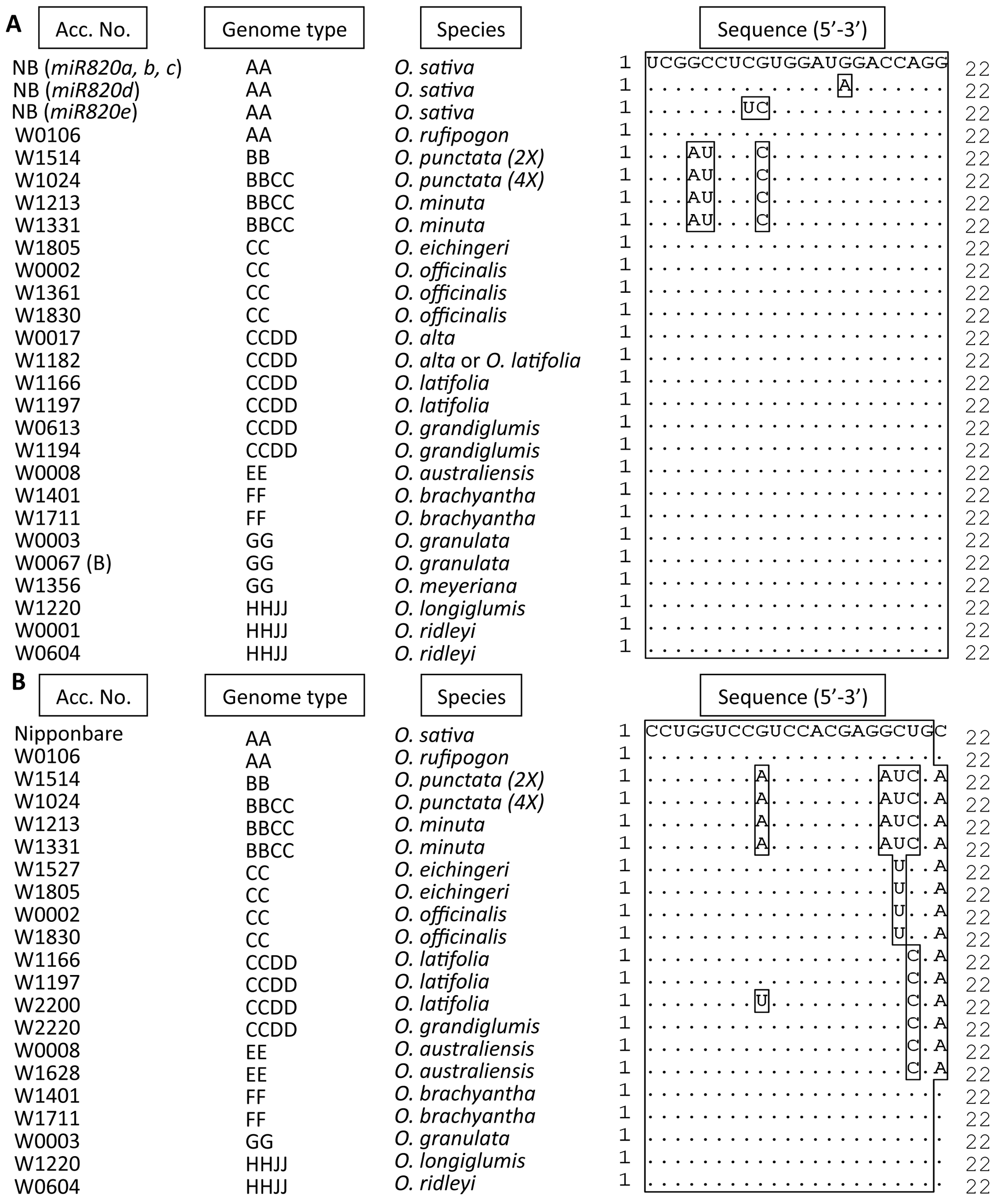

Supplement: Figure S7 — Sequence alignments of miR820 and its target site in DRM2 among Oryza species. (A) Alignment of sequences of miR820 among various Oryza species. (B) Alignment of sequences of the miR820 target site in DRM2 among various Oryza species. Dots indicate nucleotides identical to those in Nipponbare miR820a/b/c (A) or DRM2 (B). (TIF) [file pgen.1002953.s007.tif]
