# Supplementary material for: Role of Transposon-Derived Small RNAs in the Interplay between Genomes and Parasitic DNA in Rice
Source: PLoS Genet. 2012 Sep 27;8(9):e1002953. doi: 10.1371/journal.pgen.1002953 (PMC3459959; doi:10.1371/journal.pgen.1002953)
Supplement: Table S1 — Sequencing analysis of miR820 and its target site in DRM2 among various Oryza species. (DOCX) [file pgen.1002953.s008.docx]

Table S1. Sequencing analysis of *miR820* and its target site in *DRM2* among various *Oryza* species.

| Acc. No. | Genome type | Species | *miR820* sequence identity *** | *DRM2* sequence identity* | miRNA target score ** |
| --- | --- | --- | --- | --- | --- |
| Nipponbare | AA | *O. sativa* | *miR820a/b/c* | 22/22 | 2.5 |
|  |  |  | 22/22 |  |  |
|  |  |  | *miR820d* |  | 3.5 |
|  |  |  | 21/22 |  |  |
|  |  |  | *miR820e* |  | 5.5 |
|  |  |  | 20/22 |  |  |
| W 0106 | AA | *O. rufipogon* | 22/22 | 22/22 | 2.5 |
| W 1514 | BB | *O. punctata (2X)* | 19/22 | 17/22 | 2 |
| W 1024 | BBCC | *O. punctata (4X)* | 19/22 | 17/22 | 2 |
| W 1213 | BBCC | *O. minuta* | 19/22 | 17/22 | 2 |
| W 1331 | BBCC | *O. minuta* | 19/22 | 17/22 | 2 |
| W 1805 | CC | *O. eichingeri* | 22/22 | 20/22 | 2.5 |
| W 0002 | CC | *O. officinalis* | 22/22 | 20/22 | 2.5 |
| W 1830 | CC | *O. officinalis* | 22/22 | 20/22 | 2.5 |
| W 1166 | CCDD | *O. latifolia* | 22/22 | 20/22 | 0.5 |
| W 1197 | CCDD | *O. latifolia* | 22/22 | 20/22 | 0.5 |
| W 0008 | EE | *O. australiensis* | 22/22 | 20/22 | 0.5 |
| W 1401 | FF | *O. brachyantha* | 22/22 | 22/22 | 2.5 |
| W 1711 | FF | *O. brachyantha* | 22/22 | 22/22 | 2.5 |
| W 0003 | GG | *O. granulata* | 22/22 | 22/22 | 2.5 |
| W 1220 | HHJJ | *O. longiglumis* | 22/22 | 22/22 | 2.5 |
| W 0604 | HHJJ | *O. ridleyi* | 22/22 | 22/22 | 2.5 |

*The number of identical nucleotides of *miR820* and its target site in *DRM2*, respectively, between Nipponbare and various *Oryza* are shown, followed by dash and total length of each sequence.

**miRNA target score (Allen et al*.* 2005) [30] indicates the degree of the mismatches as perfect match as zero.
