# Supplementary material for: Role of Transposon-Derived Small RNAs in the Interplay between Genomes and Parasitic DNA in Rice
Source: PLoS Genet. 2012 Sep 27;8(9):e1002953. doi: 10.1371/journal.pgen.1002953 (PMC3459959; doi:10.1371/journal.pgen.1002953)
Supplement: Table S2 — Primers used in this study. (DOCX) [file pgen.1002953.s009.docx]

Table S2. Primers used in this study.

| Target gene | Primer name | Sequence (5’ to 3’) | Purpose |
| --- | --- | --- | --- |
| *OsDRM2* | qDRM1aF1 | CAGAGGTAGCTCTCCACAGAC | RT-PCR |
|  | qDRM1aR2 | CAGCTCTTCAGAATCGTTCTG | RT-PCR |
| *OsActin* | ActAK060893Fw | GAGTATGATGAGTCGGGTCCAG | RT-PCR |
|  | ActAK060893Rv | ACACCAACAATCCCAAACAGAG | RT-PCR |
| *sGFP* | sGFPF2 | AAGCTGGAGTACAACTACAAC | RT-PCR |
|  | sGFPR1 | TTGTGGCGGATCTTGAAGTTC | RT-PCR |
| *CACTA (ORF1)* | CAChprF10 | GTATGAATATGGCAAGCCGTT | RT-PCR |
|  | CAChprR10 | GAAACTGAAGGCGAAGTTTGC | RT-PCR |
| *Tos17 (TPase)* | Tos17F3 | CACCAGGTGTGGAAGCTCCAC | RT-PCR |
|  | Tos17R3 | TACCACTGAGCTGAAGCGTGC | RT-PCR |
| *RIRE7 (TPase)* | qRIRE7F2 | TCGCCAATGATCGCCTTGGTC | RT-PCR |
|  | qRIRE7R2 | AGACGATCCATCATGACCATC | RT-PCR |
| *pre-miR820* | SatofN | TTACGTTCCTTACCGATMTTG | RT-PCR |
|  | R1SATON | TGATAACGTAMGAACTACACCTCC | RT-PCR |
| *OsDRM2* (cleaved) | GeneRacer 5’ | GGACACTGACATGGACTGAAGGAGTA | 5'RACE |
|  | AK065147R2 | TTGCTCTGTATCTGTATCCCCAATCTCCTT | 5'RACE |
| *OsDRM2* (uncleaved) | qDRM2F1 | GATAGCGACAATGATAAGTTCGAG | 5'RACE |
|  | qDRM2R3 | TCCTCGTCGACTTCAAGAGTC | 5'RACE |
| *OsActin* | ActU | TCCATCTTGGCATCTCTCAG | 5'RACE |
|  | ActL | GTACCCTCATCAGGCATCTG | 5'RACE |
| *OsDRM2 mutation1* | miRJ-mutF1 | CTAAGGAACATAGATGCTCCGGGCCCCTCAACACGATTACCGCAGGATGC | GFP fusion |
|  | miRJ-mutR1 | GGAGCATCTATGTTCCTTAGGGCCGGGGCA | GFP fusion |
| *OsDRM2 mutation2* | miRJ-mutF2 | AACATAGATGCTCCTGGTCCCTCAACACGATTACCGCAGGATGC | GFP fusion |
|  | miRJ-mutR2 | GGACCAGGAGCATCTATGTTCCTTAGGGCC | GFP fusion |
| *OsDRM2 intact* PCR | DRM2CACCF6 | CACCATGGTGGACTGGGCTTCAG | GFP fusion |
|  | DRM2MIRjR4 | CGGCAGCCTCGTGGACGGACC | GFP fusion |
| *OsDRM2 mutation1* PCR | DRM2CACCF6 | CACCATGGTGGACTGGGCTTCAG | GFP fusion |
|  | DRM2miRjR4m1 | CGGTAATCGTGTTGAGGGGCC | GFP fusion |
| *OsDRM2 mutation2* PCR | DRM2CACCF6 | CACCATGGTGGACTGGGCTTCAG | GFP fusion |
|  | DRM2miRjR4m2 | CGGTAATCGTGTTGAGGGACC | GFP fusion |
| *OsDRM2* RNAi 1 | DRM1a-PstIFw | CTGCAGAGCAATTGCTTGAGTTACTTC | RNAi construction |
|  | DRM1a-XbaIRv | TCTAGACAAGTTTGCAAGCAGTTGAAC | RNAi construction |
| *OsDRM2* RNAi 2 | DRM1a-HindIIIFw | AAGCTTCAGAGCAATTGCTTGAGTTACTTC | RNAi construction |
|  | DRM1a-SmaIApaIRv | GGGCCCGGGCAAGTTTGCAAGCAGTTGAAC | RNAi construction |
| *pre-miR820* | CACTApremiRJF2 | TGATGAATATCCTTACCAATCTTG | sequencing |
|  | CACTApremiRJR6 | CATGTTTTGATCAMATGGCTAGCT | sequencing |
| *OsDRM2* | qDRM2F1 | GATAGCGACAATGATAAGTTCGAG | sequencing |
|  | qDRM2R3 | TCCTCGTCGACTTCAAGAGTC | sequencing |
| *pre-miR820* | CACTApremiRJF1 | CACATAAGAACAGACCATCTACAC | Probe amplification |
|  | CACTApremiRJR3 | TCTTTCATACTGCAATTGCGCTAG | Probe amplification |
| *CACTA (ORF1)* | HPR F | GCACAGAACCGATGTCACTAAC | McrBC-PCR |
|  | HPR R | CTTCGCCAGGTCCGACATCTTC | McrBC-PCR |
| *RIRE7 (LTR)* | RIRE7 F1 | AGGACATCCCTTCCAACGATACAAC | McrBC-PCR |
|  | RIRE7 R1 | TCTTGCCGTGCCAAGAACAACCTTG | McrBC-PCR |
| *pre-miR820* | MIRJ F | CACATAAGAACAGACCATCTACAC | McrBC-PCR |
|  | MIRJ R | ATGTTTTGATCACATGGCTAGCTC | McrBC-PCR |
| *OsActin* | ActU | TCCATCTTGGCATCTCTCAG | McrBC-PCR |
|  | ActL | GTACCCTCATCAGGCATCTG | McrBC-PCR |
| *Centromere 8* | Cen8-301 F1 | CCGATATGCCAAAGAGCGAGTC | McrBC-PCR |
|  | Cen8-301 R1 | CAAATCATCTATCCTCAAGTCC | McrBC-PCR |
| *pre-miR820* | Satof | CTTASGTTCYTTRCYGATCTTG | Phylogeny |
|  | R1SATO | TGACARCRTAYRAACTACACCTC | Phylogeny |
